# CRISPR/Cas tools for enhancing the biopreservation ability of lactic acid bacteria in aquatic products

**DOI:** 10.3389/fbioe.2022.1114588

**Published:** 2022-12-23

**Authors:** Huina Dong, Huiying Wang, Shaoping Fu, Dawei Zhang

**Affiliations:** ^1^ Tianjin Institute of Industrial Biotechnology, Chinese Academy of Sciences, Tianjin, China; ^2^ University of Chinese Academy of Sciences, Beijing, China

**Keywords:** lactic acid bacteria, aquatic products biopreservation, CRISPR/Cas system, genome editing, transcriptional regulation

## Abstract

Lactic acid bacteria (LAB) plays a crucial role in aquatic products biopreservation as it can inhibit many bacteria, in particular the specific spoilage organisms (SSOs) of aquatic products, by competing for nutrients or producing one or more metabolites which have antimicrobial activity, such as bacteriocins. *Lactobacillus* spp. and *Lactococcus* spp. are the most commonly used Lactic acid bacterias in aquatic products preservation. The improvement of gene editing tools is particularly important for developing new lactic acid bacteria strains with superior properties for aquatic products biopreservation. This review summarized the research progress of the most widely used CRISPR/Cas-based genome editing tools in *Lactobacillus* spp. and *Lactococcus* spp. The genome editing tools based on homologous recombination and base editor were described. Then, the research status of CRISPRi in transcriptional regulation was reviewed briefly. This review may provide a reference for the application of CRISPR/Cas-based genome editing tools to other lactic acid bacteria species.

## 1 Introduction

Lactic acid bacteria (LAB) are a group of Gram-positive, non-sporulating, aerotolerant bacteria characterized by their ability to produce lactic acid as the principal final product, including seven genera: *Lactococcus*, *Lactobacillus*, *Enterococcus*, *Pediococcus*, *Streptococcus*, *Leuconostoc* and *Oenococcus* (Stefanovic et al., 2017)*.* LABs have great potential for application in biological preservation and naturally dominate the microflora of many foods because most LABs are generally considered safe (Ghanbari et al., 2013).

Aquatic products are popular for their delicious taste and high nutritional value as they are rich in protein, fat, vitamins, and minerals. However, the high contents of various nutrients and moisture in aquatic products will lead to rapid microbial growth, metabolism, and biochemical reactions in *postmortem* aquatic products (Olatunde and Benjakul 2018). These fast-growing bacteria are called specific spoilage organisms (SSOs), which will become dominant as storage time increases and ultimately lead to the spoilage of aquatic products, such as *Pseudomonas* spp., *Aeromonas* spp., and *Shewanella* spp. (Zhang et al., 2019).

**FIGURE 1:**
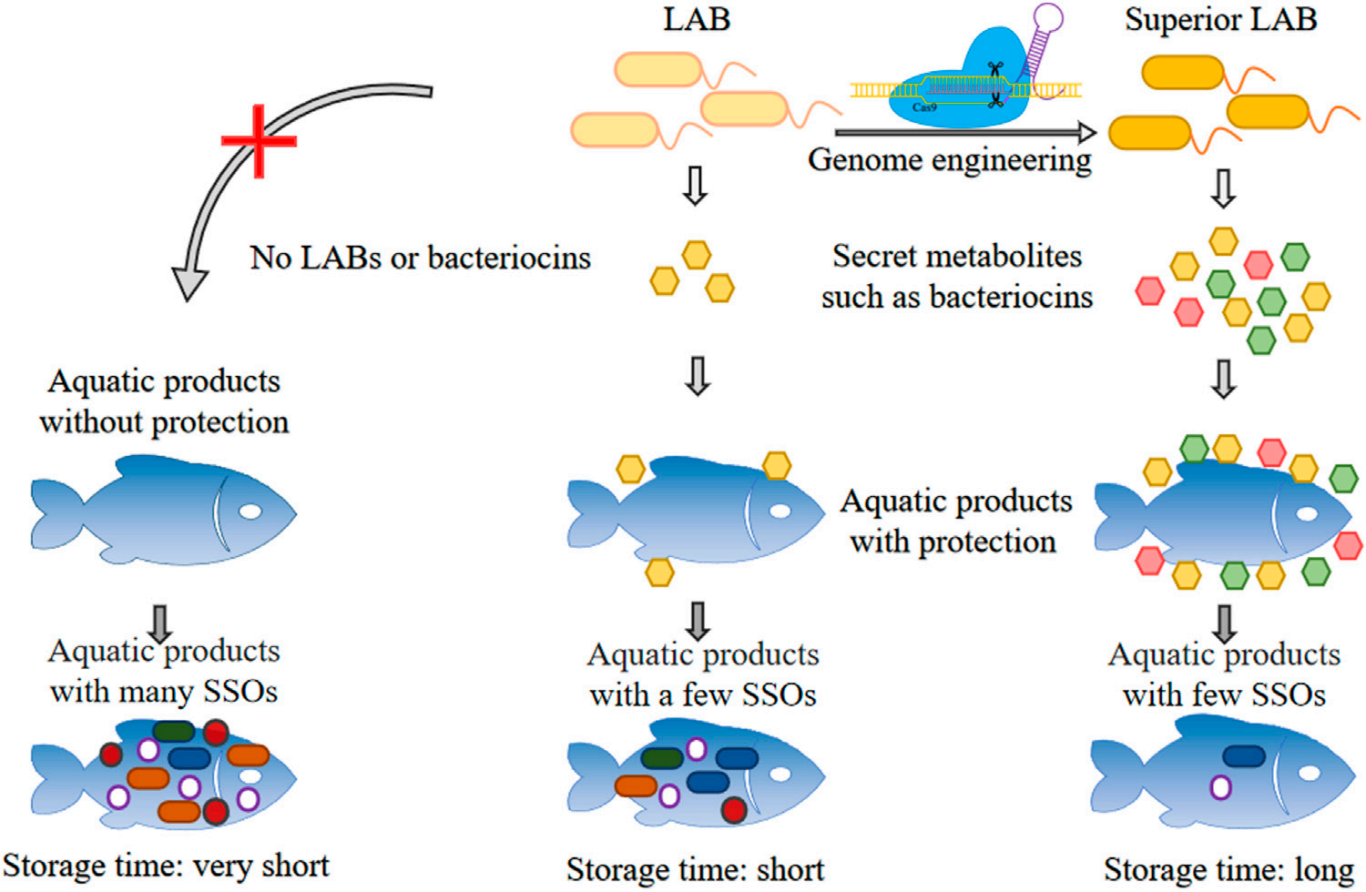
The diagram of relationship between aquatic products, SSOs and LABs. The SSOs will become dominant along with the storage time increases and lead to the spoilage of aquatic products. When the LABs or the bacteriocins produced by LABs were added to aquatic products, the shelf life of aquatic products could be extended. Improving the performance of LABs through genome editing can further extend the shelf life of aquatic products.

LABs can produce a number of bacteriocins, which are proteins or polypeptides that can be used to inhibit the growth of SSOs in aquatic products (Bali et al., 2016). The growth of LABs can also inhibit many SSOs by competing for nutrients or producing one or more metabolites with antimicrobial activity (Ghanbari et al., 2013). The most commonly used LABs in aquatic products preservation are *Lactobacillus* spp., followed by *Lactococcus* spp. (Dong et al., 2022), which are compatible with their environments, such as modified atmosphere packaging, low temperatures, and pH, and so on. The improvement of genome editing tools is particularly important for the development of new strains of LABs with excellent biopreservation properties for aquatic products (Figure 1).

Several gene-editing tools have been developed to achieve stable integration of sequences in *Lactobacillus* spp. and *Lactococcus* spp., for example, the use of insertion sequence (IS) elements (Walker and Klaenhammer 1994), Cre-*lox*-based systems (Lambert et al., 2007) and methods using selectable markers. However, the employment of IS elements is restricted by the presence and distribution of the IS sequences in the genome. The Cre-*lox*-based system will leave remnant sequences (scars) at the targeted site. And the selection markers should be eliminated in the following step for subsequent modifications and many methods using selectable markers can only be used in strains that have a specific gene mutated (Dong et al., 2022).

The genome engineering tools based on the clustered regularly interspaced short palindromic repeats (CRISPR)/CRISPR-associated protein (Cas) systems have been widely used in microorganisms. The CRISPR/Cas systems are classified into two classes (class I and class II) and six types (type I ∼ VI), and types I, III, and IV belonging to class I and types II, V, and VI belonging to class II (Mohanraju et al., 2016). Recently, many methods based on recombineering and CRISPR/Cas system had been successfully established in *Lactobacillus* spp. and *Lactococcus* spp. Here, we will summarize the development and application of CRISPR/Cas-based genome editing tools in *Lactobacillus* spp. and *Lactococcus* spp. according to different types of genome editing and transcriptional regulation scenarios. And we will discuss the different shortcomings of current CRISPR/Cas-based genome editing technologies and suggest possible directions for the future development of LABs.

## 2 Type I CRISPR/Cas-mediated genome editing

Type I CRISPR/Cas systems with Cas3 protein as hallmark can be divided into seven subtypes: I-A to I-F and I-U. Hidalgo-Cantabrana *et al* identified widespread existence of CRISPR-Cas systems in *Lactobacillus crispatus*, including type I-B, complete type I-E, and type II-A (Hidalgo-Cantabrana et al., 2019). They characterized the native type I-E CRISPR-Cas system and developed an efficient chromosomal targeting and genome editing tool based on this system with a 5′-AAA-3′ protospacer adjacent motif (PAM). In the genetic target of the *p-gtf* gene, which encodes the exopolysaccharide priming-glycosyl transferase, the 643-bp gene deletion efficiency is 100%, the stop codon insertion efficiency is 36%, and the single nucleotide substitution efficiency is 19%. However, in the prophage DNA packaging *Nu1*, the 308-bp deletion efficiency is only 20%, and the insertion efficiency of a 730-bp green fluorescent protein gene in the downstream of enolase is 23%.

## 3 Type II CRISPR/Cas-mediated genome editing

**FIGURE 2:**
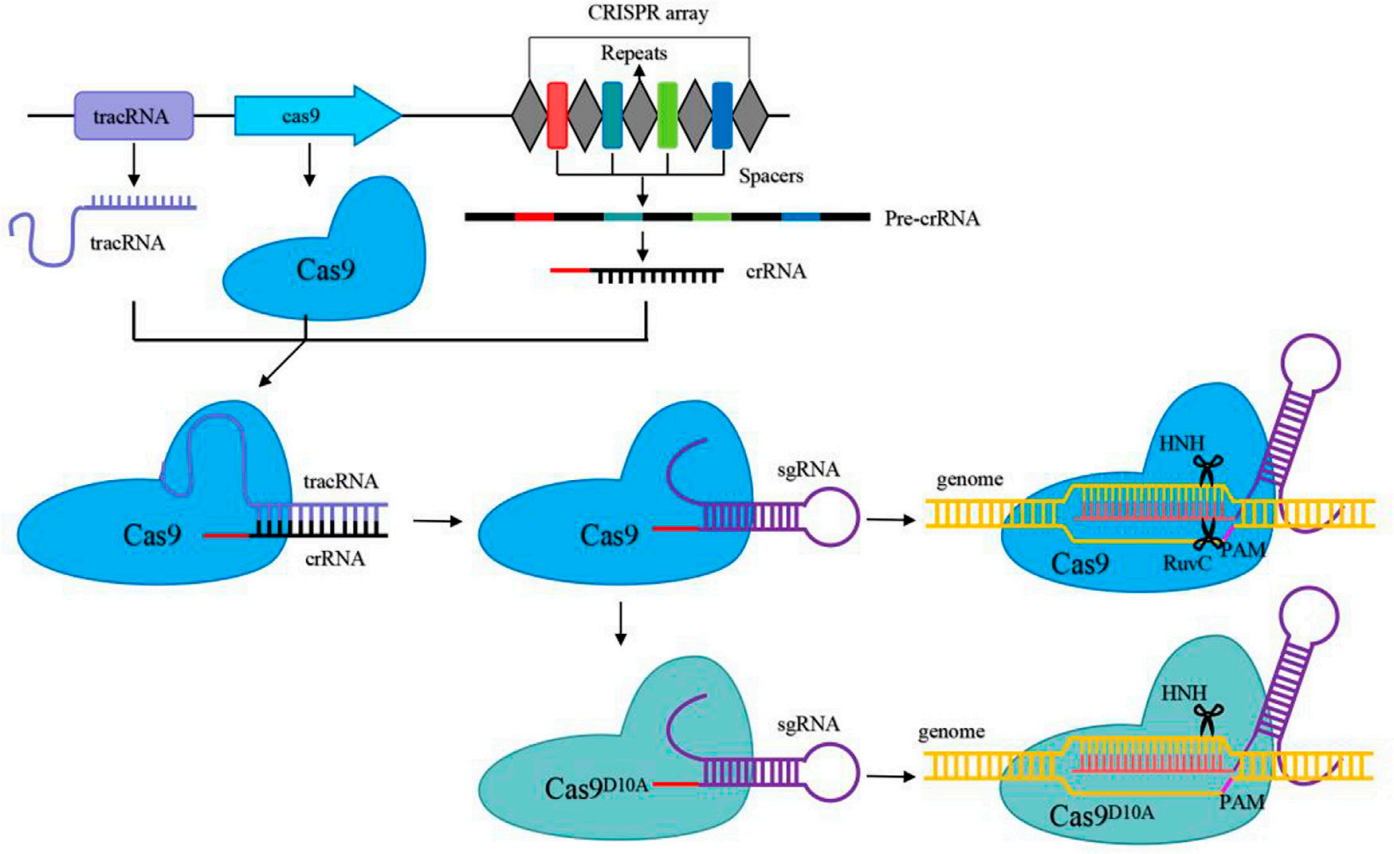
Schematic overview of the CRISPR/Cas9 and CRISPR/Cas9^D10A^ systems as genome engineering tools. Pre-crRNA is created by transcription of the CRISPR array, while mature crRNA is created by further processing of pre-crRNA. Mature crRNA binds to tracrRNA to produce guide RNA by base pairing. A connecting loop could be used to simplify the crRNA-tracrRNA chimera to form sgRNA. Then the sgRNA binds to Cas9 or Cas9^D10A^ and guides Cas9 and Cas9^D10A^ to cleave target DNA before the PAM sequence to create a double-strand break and a single-strand break, respectively.

**FIGURE 3:**
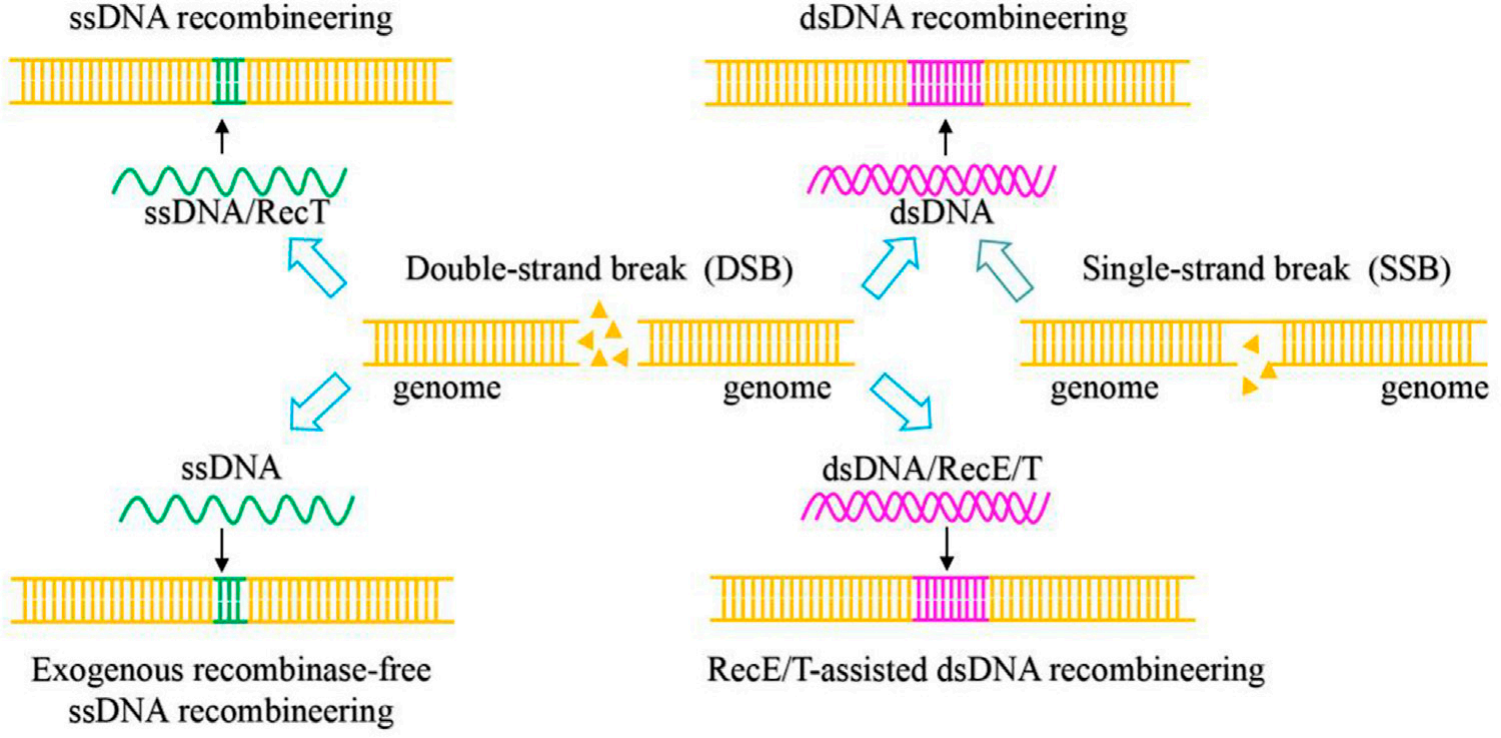
Mechanism of CRISPR/Cas9 and CRISPR/Cas9^D10A^-mediated genome editing systems based on homology-directed repair (HDR) with different repair templates.

Types II CRISPR/Cas system relies on a single effector nuclease Cas9. Cas9, which contains HNH and RuvC nuclease domains, was used as a scissor to cleave target DNA to generate double-strand breaks (Jiang and Doudna 2017). CRISPR/Cas9 is a fast-growing and powerful tool for genome engineering in various organisms, and it is the most widely used in LAB (Hidalgo-Cantabrana et al., 2019). The natural CRISPR/Cas9 system consists primarily of two components: Cas9 effector nucleases and guide RNA (gRNA), which is composed of crRNA and tracrRNA (Jinek et al., 2012; Panda and Ray 2022). The 20–24 NT bases at the 5′-end of sgRNA were used to recognize the target sequences by base pairing with target DNA with the presence of an appropriate PAM sequence (the commonly used SpyCas9 from *Streptococcus pyogenes* is 5′-NGG-3′) at the 3′-end. The CRISPR/Cas9 genome editing process is as follows: the gRNA binds to Cas9 to recruit Cas9 to the target DNA sites and activates Cas9 nuclease activity to cleave the double-strand DNA sequence to generate DSBs (Figure 2) (Anders et al., 2014). With several recombination techniques, including plasmid-assisted recombination, ssDNA and dsDNA-assisted recombination, the DSBs can be repaired by homology-directed repair (HDR) for precise gene editing (Figure 3). Besides, the DSBs can be repaired by non-homologous end joining (NHEJ) to produce inaccurate editing (Hsu et al., 2014). The DSBs in *Lactobacillus* are often repaired by HDR rather than NHEJ (Song et al., 2020).

### 3.1 Exogenous type II CRISPR/Cas-assisted recombineering

#### 3.1.1 CRISPR/Cas9-assisted ssDNA recombineering

Oh *et al.* for the first time used the CRISPR/Cas9 technology to edit the genes of *Lactobacillus* successfully (Oh and van Pijkeren 2014). They coupled CRISPR/SpCas9 with single-stranded DNA (ssDNA) recombination with the help of RecT in *Lactobacillus reuteri* for mutation target genes. RecT is used for single-stranded DNA recombineering, while CRISPR/SpCas9 plays a role in the selection of mutants. The RecT, ssDNA, Cas9, tracrRNA, and crRNA were transferred into *L. reuteri* by a dual-step approach, which could obtain a larger number of transformations than a single-step approach. The recombination rates at the *lacL*, *strA*, and *sdp6* sites could reach 90%–100%, while the mutation rate in *L. reuteri* without the aid of CRISPR/SpCas9 was only 0.4%–19% (van Pijkeren and Britton 2012). The fragments up to 1-kb could be deleted with low efficiency. Guo *et al.* selected a highly active RecT to mediate CRISPR/Cas9-assisted ssDNA recombineering in *Lactococcus lactis* (Guo et al., 2019)). The optimized system can achieve precise point mutations, seamless genomic DNA deletions (50/100 bp), and insertions (a *loxP* site, 34 bp) at efficiencies of >75%.

#### 3.1.2 CRISPR/Cas9-assisted dsDNA recombineering

Zhou *et al.* successfully accomplished seamless gene deletion and insertion in *Lactobacillus plantarum* WCFS1 using CRISPR/Cas9-assisted dsDNA recombineering (Zhou et al., 2019). The dsDNA and the plasmid, which contained Cas9 and sgRNA, were co-transformed into the cells, and the *nagB* gene (631 bp) was effectively knocked out. The 5′-end of the dsDNA was modified with thiophosphate to stop it from being cut by intracellular exonucleases and to further increase the deletion efficiency. The editing efficiency was increased more than twice, to 53.3%. And a two-step recombineering technique for gene insertion was created with the aid of the loxP/Cre system, and its efficiency is 58.3%. However, dsDNA recombineering assisted by CRISPR/Cas9 was less effective in producing point mutations than ssDNA recombineering. Vento *et al.* used two *Escherichia coli-Lactobacilli* shuttle vectors to perform genome editing in *L. plantarum* based on CRISPR/Cas9-mediated dsDNA recombineering (Vento and Beisel 2022). One vector expressed SpCas9, a single spacer CRISPR array, and tracrRNA, and the other vector contained a dsDNA editing template and homologous arm. *L. plantarum* was edited after two vectors were consecutively transformed, which can be accomplished in 10 days.

#### 3.1.3 CRISPR/Cas9^D10A^ nickase-assisted recombineering

DSBs are highly cytotoxic in some bacteria, and the repair ability of the DSBs induced by the CRISPR/Cas9 system is low. Song *et al.* replaced the wild-type Cas9 with Cas9^D10A^ to resolve the problem (Song et al., 2017). They established a rapid and precise genome editing plasmid, pLCNICK, which contains Cas9^D10A^, sgRNA, and the repair templates for *Lactobacillus casei* genome engineering. The efficiencies of deletion and insertion are 25%–62%. This genetic tool reduces the cycle time to 9 days and enables effective single-gene deletion and insertion by one-step transformation. However, when the deletion size increases, the deletion efficiency of pLCNICK falls significantly. According to reports, pLCNICK could delete gene fragments up to 3 kb in size. The isolation of mutants, which takes 2–3 days, is another drawback of pLCNICK. Goh *et al.* successfully established a single-plasmid gene editing tool (pLbCas9N) based on Cas9^D10A^ in *Lactobacillus acidophilus* (Goh and Barrangou 2021). The pLbCas9N vector harbored Cas9^D10A^, sgRNA, and an editing template. The mutant recovery rates of genome deletions between 300 bp and 1.9-kb at three loci ranged from 35% to 100%, and the deletion mutants could be recovered within a week following transformation. The pLbCas9N system was further successfully applied in *Lactobacillus gasseri* and *Lactobacillus paracasei* for generating single-base substitutions and gene deletions.

#### 3.1.4 RecE/T-assisted CRISPR/Cas9 system

RecE functions as a 5′-3′ exonuclease that cleaves exogenous double-stranded DNA to produce a 3′-ended overhang. RecT, as a single-strand annealing protein, can bind to the overhangs of single-strand DNA and facilitate strand invasion and exchange. Several bacterial species have exploited RecE/T-assisted dsDNA recombineering to increase recombineering efficiency (Binder et al., 2013; Yin et al., 2015). Yang et al. (2015) found that RecE/T also existed in *L. plantarum* and established a dsDNA recombination system to carry out homologous recombination between a heterologous dsDNA template and host genomic DNA in *L. plantarum*. Huang et al. (2019) developed a universal toolkit that combined RecE/T from phages with CRISPR/Cas9. The toolkit contains a broad-spectrum host CRISPR editing plasmid (carrying Cas9, sgRNA, and homologous arms) and a host-associated RecE/T helper plasmid to improve the effectiveness of repairs. Gene insertion is accomplished with 35.7% efficiency and gene deletion with 50%–100% efficiency in 7 days using the RecE/T-assisted CRISPR/Cas9 toolkit. This toolkit is capable of successfully editing the genomes of *L. plantarum* WCFS1 and *Lactobacillus brevis* ATCC367. But the suitable RecE/T pairing proteins should be selected in different organisms due to the fact that they are typically species-specific.

#### 3.1.5 CRISPR/Cas9-assisted exogenous recombinase-free recombineering

Exogenous recombinases serve a crucial role in recombination-based gene editing techniques, but they have also been shown to be a challenge for effective gene editing. Additionally, it has been demonstrated that co-transformation of oligos with the CRISPR/Cas9 plasmid significantly lowers transformation efficiency. Leenay et al. (2019) used an exogenous recombinase-free method for gene editing in *L. plantarum* in an effort to streamline CRISPR/Cas9-assisted ssDNA recombineering. Three genes were successfully edited after Cas9, sgRNA, and the recombination template were introduced into *L. plantarum* WJL strains, including the insertion of an early stop codon in the *ribB* gene, which codes for ribofavin synthase; the induction of several point mutations in the *ackA* gene, which codes for acetate kinase; and the complete deletion of the *lacM* gene (960 bp), which codes for a subunit of β-galactosidase. The intended mutant, however, was not produced when the *ribB* site was targeted by ssDNA-mediated recombination. Gene editing in *L. plantarum* NIZO2877 (Martino et al., 2018) and *L. paracasei* strain B (Siedler et al., 2020) was also accomplished successfully using the exogenous recombinase-free approach, but failed in *L. plantarum* WCFS1. The studies on gene editing in these three *L. plantarum* strains indicated that the effectiveness of gene editing differed according to the targeted gene and strain.

### 3.2 Endogenesis type II CRISPR/Cas-assisted recombineering

The type II CRISPR/Cas system has been constructed as a mature tool for gene editing that has been successfully applied to a number of different species. However, the application of CRISPR-Cas9 technology in LAB still has limitations, mainly because of the large size of the editing vectors, the low transformation efficiency, and the cytotoxicity of exogenous Cas9. A large number of endogenous CRISPR/Cas components were found on the LAB genome (Sanozky-Dawes et al., 2015; Song et al., 2020). Crawley et al. (2018) discovered that type II-A systems in *Lactobacilli* were naturally active in their hosts, expressing themselves and effectively destroying invading and genomic DNA. They can be completely leveraged to overcome the difficulties of the existing editing systems in the creation of genome editing tools, which have the advantages of ease of transformation due to the relatively tiny targeting vector and no concern about the toxicity of heterologous Cas9 to host cells. It could be more appropriate for editing the genome of LAB and for some LAB it might eventually become the main genome editing tool.

## 4 Type III CRISPR/Cas-mediated genome editing

The type III CRISPR/Cas systems can be classified into four subtypes A-D. The Type III-A system is one of the most widely distributed CRISPR/Cas systems across prokaryotic phyla and cleaves DNA and RNA molecules.

The III-A CRISPR/Cas module from *L. lactis*, which include a Cas6 protein, a CRISPR locus for crRNA production, and Csm effector complex proteins, was heterologously expressed in *E. coli* (Ichikawa et al., 2017). The expressed module specifically eliminated an invasive plasmid recognized by the crRNA. When appropriate crRNA sequences were added to the module, the module could be programmed to recognize plasmids with novel target sequences. This system lays the foundation for developing it as a gene editing tool in *E. coli* or other novel organisms*.*

## 5 CRISPR/Cas-mediated transcriptional regulation

**FIGURE 4:**
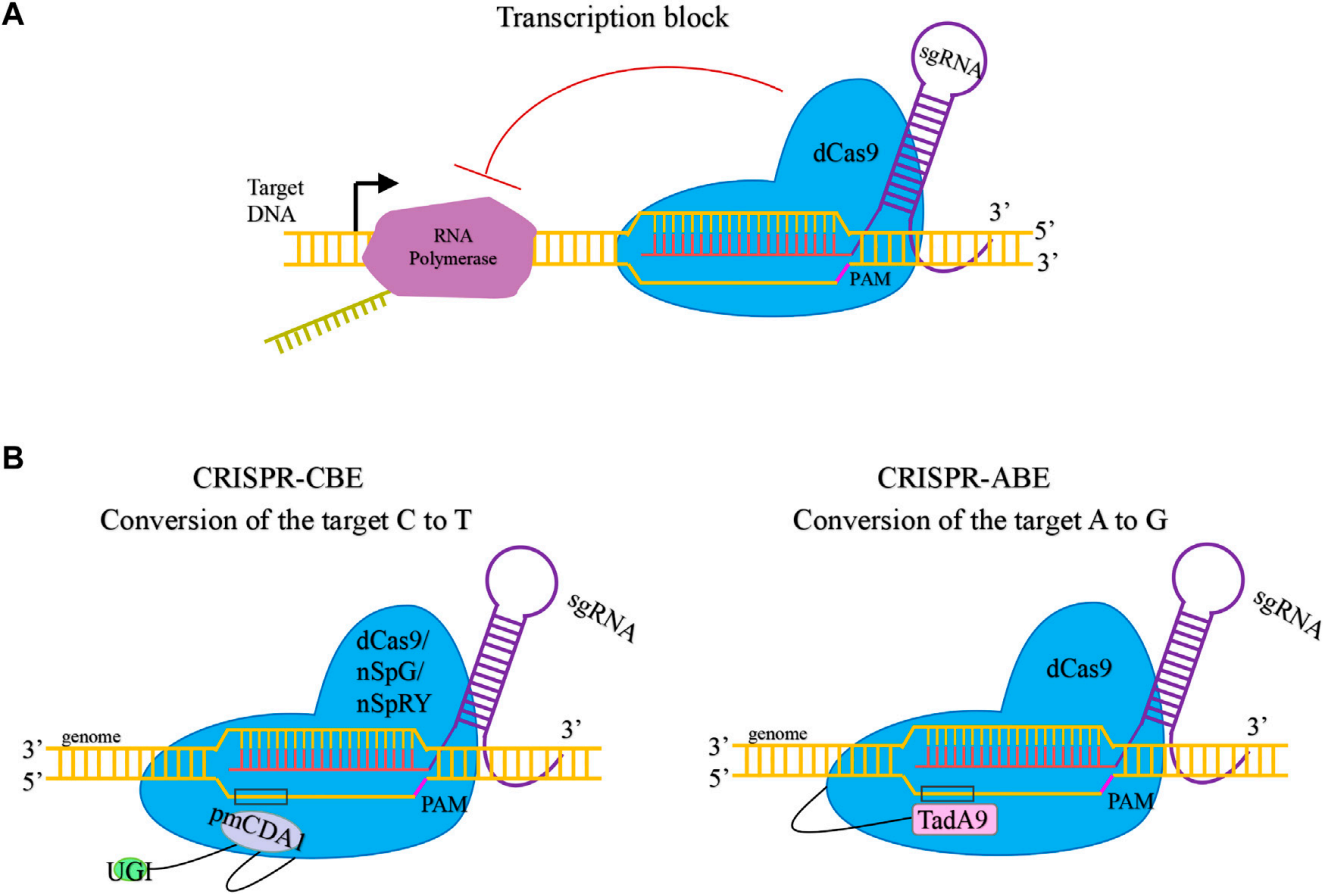
Mechanisms of dCas9-mediated transcriptional regulation and base editor. **(A)**. Transcription block by dCas9. **(B)**. Base edition by dCas9-mediated CBE and ABE.

Replacing Cas9 in the CRISPR/Cas9 system with dCas9 enabled CRISPR interference-mediated silencing of genes (Figure 4A). Berlec *et al* used CRISPRi to mediate the *upp* gene silencing in *L. lactis*, which decreased *upp* mRNA transcription and prevented the toxicity of 5-fuorouracil (Berlec et al., 2018). Xiong *et al* constructed a two-plasmid CRISPRi system in *L. lactis* (Xiong et al., 2020)*.* The dCas9 was expressed under inducible promoter P_nisin_ and in one plasmid and the sgRNA for single or multiple target genes was expressed under a strong constitutive promoter P_44_ and expressed in the other plasmid. This system mediated silencing of single or multiple genes significantly reduce gene expression by up to 99%.

Myrbraten et al (2019) developed a similar two-plasmid CRISPRi system for knockdown of gene expression in *L. plantarum*, in which the dCas9 and sgRNAs are expressed on separate plasmids. The CRISPRi system was used to preliminarily understand the functions of many key cell cycle genes in *L. plantarum.*

## 6 CRISPR/Cas-mediated base editor

Although the CRISPR/Cas systems have been developed for robust genetic manipulations, base editors can simultaneously perform genome editing at multiple endogenous loci without inducing DSBs (Figure 4B). Tian et al (2022) successfully established the Cytosine-to-Thymine base editor (CBE) and Adenine-to-Guanine base editor (ABE) in *L. lactis* by combining dCas9 and deaminase*.* CBE can be used to simultaneously inactivate multiple genes using a single plasmid. Continuous gene editing by CBE and ABE could be achieved by using temperature-sensitive plasmids, which can be cured quickly. It was found that Cas9 variants SpG and SpRY can expand the editing scope of gene inactivation in *L. lactis*, and the preference of these variants for the PAM in *L. lactis* was evaluated.

## 7 Disscussion and future prospects

Natural preservatives like bacteriocins can effectively replace synthetic preservatives because of their excellent antimicrobial and antioxidant properties. However, the low yield and the high cost of production restricted their application as biopreservatives in aquatic products. The introduction of acid-tolerant genes or over-expression of lactic acid synthesis pathway genes can increase the production of bacteriocins by LABs (Zhang et al., 2016). The bacteriocin production levels could also be increased by increasing the carbon conversion rates in the central pathway under oxidizing conditions through the expression of relevant genes (Papagianni and Avramidis 2012). LABs as biological preservatives in aquatic products have been explored in recent years as they can inhibit the growth of spoilage bacteria to prolong the shelf -life of aquatic products. However, some LAB metabolites may influence the sensory characteristics of aquatic products. Genome engineering can be used to improve the production of natural preservatives such as bacteriocins and the performance of LABs.

*Lactobacillus* or *Lactococcus* strains are widely used in aquatic products biopreservation, and it is important to develop them with excellent performance. However, conventional gene-editing methods of *Lactobacillus* or *Lactococcus* strains are inefficient. For example, the employment of insertion sequence (IS) elements is restricted by the presence and distribution of IS sequences in the genome that have low genetic stability; the employment of Cre-lox-based systems will leave remnant scars at the targeted site; and other systems using selectable markers (such as antibiotic resistance genetic markers that result in low biosafety) can only be used in strains that have a specific gene mutated. Compared with conventional gene-editing systems, the CRISPR/Cas9-based system was easy to operate, trace-free, and had high genetic stability and biosafety.

Recently, the technique of producing superior *Lactobacillus* or *Lactococcus* strains through CRISPR/Cas-based genome editing has attracted a large number of researchers’ interest. Currently, the most commonly used Cas protein in *Lactobacillus* or *Lactococcus* is Cas9. CRISPR/Cas9 mediated genome editing in *Lactobacillus* or *Lactococcus* strains can be mainly divided into three types according to their working principles. One type is CRISPR/Cas9-mediated double-stranded breaks, endogenous or exogenous recombinases-mediated ssDNA or dsDNA recombination to facilitate gene editing, and CRISPR/Cas9 to eliminate unedited cells. The other type is CRISPR/nCas9-mediated single-stranded breaks, where endogenous or exogenous recombinases are used to repair the breaks. Another type is CRISPR/dCas9-mediated gene targeting with double-strand break-free, cytidine and adenosine deaminases to make target base editing. Furthermore, dCas9 itself can be used to suppress gene transcription.

The application of CRISPR/Cas-based genome editing to improve *Lactobacillus* or *Lactococcus* for aquatic product biopreservation may be a promising method. Genome editing of *Lactobacillus* or *Lactococcus* strains is now approaching the goal of increasingly efficient editing and lower off-target rates, but it is important to see the limitations of CRISPR/Cas technology, such as the cytotoxicity of Cas9 to some *Lactobacillus* or *Lactococcus* strains. Many CRISPR/Cas systems perform well in model strains but not in non-model strains. It is difficult to find a universal gene editing tool for all *Lactobacillus* or *Lactococcus* strains. Therefore, the development of diverse gene-editing tools with complementary editing strategies is essential.

A series of CRISPR/Cas gene editing systems were developed to allow for wide application. Anzalone et al. (2019) developed a Prime Editor for precision gene editing. They used CRISPR/Cas9 and reverse transcriptase to effectively create all forms of base alterations without DNA templates and DSBs, as well as precise multiple base insertion and deletion. Solving the problem of inserting large gene fragments with recent tools is difficult. Strecker *et al.* built a CRISPR/Cas-based transposase system that consists of Cas12k (a Type V-K CRISPR effector with a naturally inactivated RuvC-like nuclease domain) and a Tn7-like transposase (Strecker et al., 2019). This system could integrate ∼10 kb of DNA fragments into the *E. coli* genome without positive selection and generating DSBs. Furthermore, Sternberg *et al.* reported a type I-F CRISPR/Cas-based Tn7-like transposon system to insert DNA fragments into the *E. coli* genome that is more efficient than homologous recombination-mediated gene insertion (Klompe et al., 2019). And there are many other Cas proteins widely used in other microorganisms, such as Cas12a, which can also be used in *Lactobacillus* or *Lactococcus,* all of which can mediate DSB.

Except for the exogenous CRISPR/Cas system, many of the *Lactobacillus* or *Lactococcus* strains possessed one or more endogenous CRISPR/Cas systems, which could be exploited for genome editing after developing an appropriate gRNA. These CRISPR systems offer novel prospects for gene editing in strains of *Lactobacillus* or *Lactococcus*, and they are anticipated to be utilized in future studies.
